# Transcolonoscopic spraying formalin solution for hemorrhagic radiation proctitis: a retrospective analysis

**DOI:** 10.3389/fmed.2023.1241833

**Published:** 2024-01-05

**Authors:** Kun Huang, Xiaolin Zhao, Jiufei Yu, Jianping Cheng, Lili Wu

**Affiliations:** ^1^Department of Gastroenterology, Civil Aviation General Hospital, Beijing, China; ^2^Department of Gastroenterology, The Second Medical Center, Chinese People’s Liberation Army General Hospital, Beijing, China

**Keywords:** hemorrhagic radiation proctitis, formalin, colonoscopy, bleeding, spraying

## Abstract

**Background:**

Radiation proctitis is a common complication that occurs as a result of radiation therapy used to treat pelvic malignancies. The most common and bothersome symptom resulting from radiation proctitis is rectal bleeding, which can be persistent or recurrent. This study aimed to review our experience and evaluate the efficacy and safety of transcolonoscopic spraying of formalin solution in patients with hemorrhagic radiation proctitis.

**Methods:**

A total of 37 patients with hemorrhagic radiation proctitis, aged between 48 and 79 years (mean age 62.56 ± 8.48 years), were divided into three cohorts based on the severity of radiation injury. Under direct endoscopic vision, a 4% formalin solution was applied directly to the rectal hemorrhagic mucosa. The patients were followed for a period of over 6 months after receiving treatment, during which the therapeutic effectiveness and occurrence of complications were observed.

**Results:**

The study resulted in an overall response rate of 89.2% among all patients. The response rates for patients with grades 1–3 were 100, 100, and 66.7%, respectively. Notably, the rate of response among patients with grade 3 radiation injury was significantly lower compared to those with grades 1–2 (*p* = 0.009). Mild adverse reactions, such as anal pain and tenesmus, were reported in a small number of patients but could be alleviated without any intervention.

**Conclusion:**

The endoscopic application of formalin solution for the treatment of hemorrhagic radiation proctitis has shown a significant effect, particularly in patients with grades 1–2 radiation injury. The observed effect is superior to that observed in patients with grade 3 radiation injury.

## Introduction

Radiation proctitis is a frequently encountered complication arising from radiation therapy administered for pelvic malignancies. This is primarily attributed to the anatomical positioning and histological attributes of the rectum, particularly in cases of prostatic and cervical cancer (1, 2). The reported incidence of radiation proctitis can be as high as 2–5% (3). This condition predominantly manifests following the conclusion of radiation therapy, possibly attributable to the detrimental effects of ionizing radiation-generated free radicals on the cells of the rectal mucosa. The pathological consequences encompass various alterations, such as the suppression of enterocyte proliferation, damage to the arterioles located beneath the rectal mucosa, the development of chronic fibrosis within the rectal wall, focal deformities, and intimal fibrosis of small arteries, all of which can be observed through microscopic examination. These changes can present themselves in the form of various symptoms, including telangiectasia, stenosis, ulcers, and fistulas (4, 5). One of the most bothersome symptoms of radiation proctitis is persistent or recurrent rectal bleeding, which lacks standardized treatment approaches. The initial acute reaction usually occurs within 3 months after radiotherapy, while chronic complications such as bleeding, pain, tenesmus, diarrhea, stenosis, or fistulation may appear months or years later. In severe cases, blood transfusion may be necessary. The treatment options commonly used for radiation proctitis encompass a range of therapeutic methods, such as topical corticosteroids, 5-aminosalicylic acid, pentoxifylline or sucralfate enemas, hyperbaric oxygen treatment, and endoscopic therapy involving argon laser or heater probes (among others). Surgery is considered a last resort and is only performed in life-threatening rectal hemorrhage situations (6–8). Furthermore, technological advances, which concern both the use of highly conformal image-guided radiotherapy techniques and the personalization of target delineation to minimize unnecessary radiation delivery to the rectum, together with the early application of some topical drugs, could be helpful for preventing the onset of proctitis (9, 10).

Formalin therapy was first employed in the treatment of hemorrhagic cystitis in 1969, and subsequently investigated as a potential treatment for radiation-induced hemorrhagic proctitis in 1986. Since then, sporadic reports have appeared in the literature describing more patients and more advanced methodologies for the application of formalin in the rectum. As a sclerosing agent, formalin facilitates the chemical cauterization of mucosal vessels, with various techniques available for its application on rectal lesions. Different methods of formalin therapy have been reported in the literature. The success rate of this method in patients with radiation proctitis, in terms of cessation of bleeding, ranges from 60 to 100% (11–14). Nonetheless, there is a lack of consensus regarding the optimal patient selection and timing for formalin therapy. Moreover, the potential toxic side effects of inappropriate formalin therapy warrant caution. The application of formalin solution through direct visualization with a colonoscope allows for precise targeting of the affected lesion. This step is essential for minimizing harm to the surrounding healthy tissue and enhancing safety, thereby reducing the occurrence of severe adverse reactions. The objective of our study was to conduct a comprehensive review of our experience and assess the effectiveness and safety of transcolonoscopic formalin solution spraying in managing hemorrhagic radiation proctitis (HRP).

## Methods

### Patients

Thirty-seven hemorrhagic radiation proctitis in patients admitted to the Civil Aviation Hospital and PLA General Hospital from January 2018 to June 2022 who were treated with transcolonoscopic spraying formalin solution were enrolled in this study.

All patients had clinical evidence of radiation-induced proctitis, which was graded according to the Radiation Therapy Oncology Group/European Organization for Research and Treatment of Cancer (RTOG/EORTC) scale and the Radiation Therapy Oncology Group’s Modified Radiation Toxicity Scale (MRTS), as shown in Table 1 (15, 16). The data collected included patients’ gender, type of malignancy, other treatments before formalin use, antiplatelet or anticoagulant use, number of formalin treatments, patient tolerance, response to therapy, complications, and duration of follow-up. The inclusion criteria were (1) previous radiotherapy for pelvic tumors; (2) colonoscopy showed a radiation toxicity grade of 1–3 according to MRTS; (3) treatment with 4% formalin solution for the treatment of HRP. The exclusion criteria were (1) a radiation toxicity grade of 4 and above; (2) the possibility that the bleeding was caused by other diseases that cannot be ruled out; and (3) incomplete treatment records.

**Table 1:** Radiation therapy oncology group’s modified rectal toxicity scale (MRTS).

Grade	Clinical	Symptoms and interventions
0	No impact	No discernable symptoms or intervention
1	Mild and self-limiting	Minimal, infrequent bleeding or clear mucus discharge, rectal discomfort not requiring analgesics, and loose stools not requiring medications
2	Managed conservatively, lifestyle (performance status) not affected	Intermittent rectal bleeding not requiring regular use of pads, erythema of rectal lining on proctoscopy, and diarrhea requiring medications
3	Severe, affects patient lifestyle	Rectal bleeding requiring regular use of pads and minor surgical intervention, rectal pain requiring narcotics, and rectal ulceration
4	Life-threatening and disabling	Bowel obstruction, fistula formation, bleeding requiring hospitalization, and surgical intervention required
5	Death	Death directly related to radiation effects

The participants in this study were required to provide written informed consent before undergoing endoscopy. The study was approved by the Ethical Committee of Civil Aviation General Hospital and met the guidelines of the local responsible governmental agency.

### Treatment procedures

Bowel preparation was performed for all patients prior to the procedure. Subsequently, a routine electronic colonoscopy (CV-290, Olympus Optical Co. Ltd., Tokyo, Japan) was conducted to rule out other hemorrhagic diseases, such as colon polyps, tumors, inflammatory bowel disease, and vascular malformation. All procedures were conducted using an electronic colonoscope in the left lateral decubitus position. The position could be adjusted to either the lateral or prone position if necessary to accurately determine the location of the lesion. Furthermore, 4% formalin solution was directly sprayed on the rectal hemorrhagic mucosa by the colonoscope for 3 min until the bleeding stopped. A saline solution was then used to adequately irrigate the rectum. This procedure could be repeated once or twice if bleeding does not stop, and the total contact time between formalin and the lesion should not exceed 10 min.

### Follow-up

All the patients were monitored for blood cell counts, liver and kidney functions, and coagulation function. Patients were reviewed at the outpatient clinic 1, 3, and 6 months after discharge. They were then regularly followed up through telephone and outpatient visits, with rebleeding as the endpoint of the follow-up. Bleeding per rectum, bowel movements, common complications, and systemic toxicities (such as symptoms of respiratory irritation, abnormal liver and renal function, and pancytopenia) were recorded.

Three categories of response were evaluated: a complete response, characterized by the absence of any further episodes of bleeding within a 6-month period; a significant response, indicated by a substantial reduction in bleeding, with no more than 3 episodes occurring within 6 months, and no significant alteration in hemoglobin levels; and a failed response, denoting the persistence of bleeding despite treatment. The response rate was defined as the percentage of patients who achieved a complete response or significant response, out of the total number of patients. The complete response rate is defined as the percentage of patients who achieved a complete response out of the total number of patients.

### Statistical analysis

Data were analyzed using the SPSS 22.0 software package. The measurement data of normal distribution was described by mean ± standard deviation ($\bar{x}$± *s*), and the independent sample *t*-test was used for comparison between groups; the count data were represented by the use case (%). χ^2^ test was used for the comparison between groups. *p* < 0.05 indicates that the difference was statistically significant.

## Results

### Demographic characteristics of the patients

A total of 37 participants were included in the study, consisting of 9 men and 28 women. The gender ratio was approximately 1:3.1. The age range of the participants was 48 to 79 years, with a mean age of 62.56 ± 8.48 years. The primary tumors consisted of nine cases of prostate cancer, one case of bladder cancer, five cases of endometrial cancer, one case of vaginal cancer, and 21 cases of cervical cancer. The time period between the completion of radiotherapy and the occurrence of hemorrhagic radiation proctitis in all patients ranged from 9 to 15 months. The duration of hemorrhage prior to treatment ranged from 19 days to 13 months. Among the patients, five were severely anemic, and two required blood transfusions due to severe rectal bleeding. None of the patients underwent endoscopic therapy or received enemas, such as argon plasma coagulation (APC) or sucralfate enema, among others. The patients had no prior history of using antiplatelet and anticoagulant medications before undergoing treatment.

### Therapeutic effect of patients with various grades

The patients were categorized into three groups based on the severity of radiation-induced injuries. There was no significant difference in the age of the patients and the treatment times. The overall response rate for all patients was 89.2%. There was a significant difference in the effect of formalin on patients with different grades. The response rates observed among patients with grades 1–3 were 100, 100, and 66.7%, respectively. The response rate of patients with grade 3 was significantly lower than that of grades 1–2, as shown in Table 2.

**Table 2:** Comparison of therapeutic effect among different grades.

		Grade 1	Grade 2	Grade 3	*F/χ* ^2^	*p-*value
*n*		10	15	12		
Age ($\bar{x}$ ± *s*)		61.1 ± 7.43	62.33 ± 10.77	64.08 ± 6.17	0.334	0.718
Number of formalin treatments ($\bar{x}$±*s*)		1.8 ± 0.63	1.93 ± 0.7	2.5 ± 0.79	3.125	0.057
Response rate		100%	100%	66.7%	9.343	0.009
	Complete response	10	13	2		
	Significant response	0	2	6		
	Failed response	0	0	4		

### Complications of patients with various grades

All patients were treated with transcolonoscopic spraying of formalin, and the overall rate of adverse reactions was 16.2%. Mild adverse reactions were reported, most commonly anal pain and tenesmus, but in most cases, these symptoms resolved spontaneously. No significant complications were observed, as indicated in Table 3.

**Table 3:** Comparison of complications among different grades.

	*n*	Anal pain	Tenesmus	Fecal incontinence	Anal stenosis	Fistula	Systemic toxicities
Grade 1	10	0	0	0	0	0	0
Grade 2	15	2	1	0	0	0	0
Grade 3	12	3	3*	0	0	0	0

^*^The patient had concurrent anal pain.

## Discussion

HRP is a serious condition that can occur after radiation therapy for pelvic tumors. This condition has a profound impact on the overall quality experienced by affected patients. Digestive endoscopy holds significant importance in the diagnosis of radiation proctitis, and its technique also offers potential for therapeutic intervention. Endoscopic thermal methods are employed with the objective of eradicating all telangiectasia in order to mitigate bleeding. Currently, the use of endoscopic argon plasma coagulation is widespread, as it has been proven to be an effective and popular treatment option for patients with refractory HRP. The technique targets areas of bleeding or telangiectasia, with a success rate ranging from 83 to 100%. However, this procedure can lead to various complications, including rectal ulceration, stricture, bowel perforation, and rectovaginal fistulas (17–20).

Formalin, as a type of fixing agent, exhibits a potent protein coagulation effect. This effect leads to the precipitation of cellular proteins and the blockage of telangiectatic lesions and small capillaries. The primary site of action for formalin is the superficial layer, without extending beyond the mucosal layer. A number of studies have shown that patients can achieve a relatively high remission rate by using various concentrations of formaldehyde solution through retention enema, local perfusion, gauze infiltration, etc. (12, 21–23). However, the results of different research studies vary significantly, and the lack of consistency in analyzing methods hinders the summarization of diagnosis and treatment experience. In the present study, we conducted a retrospective analysis of our experience involving 37 patients who were treated with a 4% formalin solution under endoscopic guidance. The overall success rate reached 89.2%. Furthermore, our research has revealed variations in the therapeutic efficacy among patients with different grades of radiation-induced injuries. Given that the treatment durations were essentially equal, it was observed that the complete response rate among patients with grade 1 could reach 100%, while for grade 2, it showed a decrease. Among patients with grade 3, the response rate was found to be 66.7%, with a complete response rate of only 16.7%. Furthermore, relevant guidelines advise against the use of formaldehyde therapy in patients with radiation injury of grade 3 or higher (7). Our results suggest that formalin therapy may be more likely to achieve satisfactory therapeutic effects in patients with grade 1–2 radiation injuries.

No severe complications related to the formalin treatment were observed in this study. Only a small number of patients experienced mild symptoms, such as anal pain and tenesmus, which could resolve spontaneously. It has been reported in the literature that the administration of formaldehyde treatment may lead to various adverse effects, including dyschezia, fecal incontinence, anal stenosis, fistula, and rectal cancer (11, 24–27). There may be a certain relationship between the application of formaldehyde. Spraying a formalin solution to treat hemorrhagic radiation proctitis under direct vision with the colonoscope can accurately target the lesion. After the treatment, it is important to completely remove any residual formalin liquid. This will help reduce damage to the surrounding normal tissue and increase safety, ultimately minimizing the occurrence of serious adverse reactions. Compared to enemas, which can easily lead to proctostenosis, this treatment provides local formalin therapy under direct vision using a colonoscope. This approach helps to avoid complications associated with enemas. However, it is important to remain vigilant for the occurrence of perforated ulcers. Therefore, the quantity and duration of formalin contact are crucial factors to consider. In addition, the potential systemic toxicity of formaldehyde was also assessed in this study. After a follow-up period of more than 6 months, no systemic toxic reactions, such as pancytopenia or impairment of liver and kidney function, were observed. It is further proven that this treatment method has a high level of safety.

However, our study has several limitations that may have introduced potential bias. These limitations include the retrospective nature of the study, the small sample size, differences in disease progression, and variations in tumor treatment regimens. Wider prospective randomized controlled trials are required in order to validate the effectiveness and safety of this therapeutic approach.

## Conclusion

In conclusion, the application of formalin solution to treat hemorrhagic radiation proctitis under direct vision with the colonoscope has been found to have a significant impact. It offers convenience in terms of application, is cost-effective, and ensures a high level of safety. Especially for patients with grade 1–2 radiation injury, the treatment outcome is better than that for patients with grade 3.

## Data availability statement

The raw data supporting the conclusions of this article will be made available by the authors, without undue reservation.

## Ethics statement

The studies involving humans were approved by the Ethical Committee of Civil Aviation General Hospital. The studies were conducted in accordance with the local legislation and institutional requirements. The participants provided their written informed consent to participate in this study. Written informed consent was obtained from the individual(s) for the publication of any potentially identifiable images or data included in this article.

## Author contributions

KH, XZ, and LW: conceptualization and writing — review and editing. KH and LW: methodology and resources. KH, XZ, JY, and JC: formal analysis and investigation. KH: writing — original draft preparation. LW: supervision. All authors reviewed and approved the manuscript.
